# One size does not fit all – Trehalose metabolism by *Clostridioides difficile* is variable across the five phylogenetic lineages

**DOI:** 10.1099/mgen.0.001110

**Published:** 2023-09-28

**Authors:** Andrew Marshall, John W. McGrath, Molly Mitchell, Séamus Fanning, Geoff McMullan

**Affiliations:** ^1^​ School of Biological Sciences, Queen’s University Belfast, 19 Chlorine Gardens, Belfast, BT9 5DL, UK; ^2^​ University College Dublin-Centre for Food Safety University College Dublin, Dublin, Ireland

**Keywords:** carbohydrates, CDI, *Clostridioides difficile*, metabolism, nutrients, phylogenetic clades, trehalose

## Abstract

*
Clostridioides difficile *, the leading cause of antibiotic-associated diarrhoea worldwide, is a genetically diverse species which can metabolise a number of nutrient sources upon colonising a dysbiotic gut environment. Trehalose, a disaccharide sugar consisting of two glucose molecules bonded by an α 1,1-glycosidic bond, has been hypothesised to be involved in the emergence of *C. difficile* hypervirulence due to its increased utilisation by the RT027 and RT078 strains. Here, growth in trehalose as the sole carbon source was shown to be non-uniform across representative *C. difficile* strains, even though the genes for its metabolism were induced. Growth in trehalose reduced the expression of genes associated with toxin production and sporulation in the *C. difficile* R20291 (RT027) and M120 (RT078) strains *in vitro*, suggesting an inhibitory effect on virulence factors. Interestingly, the R20291 TreR transcriptional regulatory protein appeared to possess an activator function as its DNA-binding ability was increased in the presence of its effector, trehalose-6-phosphate. Using RNA-sequencing analysis, we report the identification of a putative trehalose metabolism pathway which is induced during growth in trehalose: this has not been previously described within the *C. difficile* species. These data demonstrate the metabolic diversity exhibited by *C. difficile* which warrants further investigation to elucidate the molecular basis of trehalose metabolism within this important gut pathogen.

## Data Summary

Whole genome sequencing data have been deposited in the BioProject database (https://www.ncbi.nlm.nih.gov/bioproject) with accession code PRJNA975498. *C. difficile* TL178 genome accession BioSample SAMN35327655, *C. difficile* CD305 genome accession BioSample SAMN35327656. RNA-sequencing data can be found at the NCBI Gene Expression Omnibus (GEO) (https://www.ncbi.nlm.nih.gov/geo/) under accession number GSE232033.

**Impact Statement**

*
Clostridioides difficile* is an opportunistic pathogen which exploits unoccupied nutrient niches following disruption of the gut microbiota during antibiotic therapy. Understanding what specific nutrient sources are used during this process would delineate their importance for successful *C. difficile* infection. Phenotypic heterogeneity exists across the *C. difficile* species, however the genetic and regulatory basis of these fundamental processes are usually addressed in a single strain, excluding important differences that may occur. Trehalose has been implicated in the emergence of *C. difficile* hypervirulence; this work highlights its variable utilisation by growth and gene expression level changes across a panel of representative strains from each of the five toxigenic clades. Importantly, we report that trehalose utilisation reduced expression of toxin and sporulation associated genes in hypervirulent strains. Furthermore, the TreR regulatory protein binds to the *treR* and *treA* trehalose metabolism genes, which may act as an activator. Finally, RNA-sequencing analysis uncovered the presence and induction of a putative trehalose metabolism pathway previously unreported in *C. difficile* trehalose metabolism. These findings underscore the importance of investigating metabolic processes across a number of *C. difficile* strains, cautioning the use of single strains to draw representative conclusions.

## Introduction

The human diet is typically composed of a vast array of macromolecules, the variation of which has a substantial impact on the structure and function of the gut microbiota [1]. Colonisation resistance, the ability of the gut microbiota to exclude pathogenic bacteria by occupying physical space, producing inhibitory compounds and outcompeting them for nutrients [2] is important in preventing *C. difficile* colonisation, and hence *C. difficile* infection (CDI) [3]. CDI is categorised as an ‘urgent threat’ to global health by the CDC as it carries significant clinical, social and economic burdens [4, 5]. CDI is caused by production of the major toxins TcdA and TcdB by *C. difficile* cells which ultimately damage the host epithelium and the induction of a pro-inflammatory environment [6]. The symptoms of CDI range from mild diarrhoea to more severe pseudomembranous colitis and in some cases death [7]. Although CDI can be treated with antibiotics, recurrent infections can occur in 20–30 % of cases due to reinfection or relapse [8].

Classically colonisation resistance towards *C. difficile* was thought to be mainly facilitated by specific gut microbiota species that convert primary bile acids to secondary bile acids thereby preventing vegetative growth [9]. Antibiotic treatment can subsequently eliminate these gut microbiota species thus enriching primary bile acids essential to activate the germination and outgrowth of *C. difficile* spores [9, 10]. However competition for nutrients, specifically the amino acid proline, by the gut microbiota is now thought to have more importance in protecting against *C. difficile* colonisation [11]. Additionally, the loss of key gut microbiota species in a dysbiotic gut is thought to allow *C. difficile* to readily capitalise on newly available nutrients and occupy the vacant niche [12–14].

*C. difficile* as a species exhibits vast metabolic flexibility, being able to utilise a range of nutrient sources to sustain its strict anaerobic lifestyle [15]. Amongst these are carbohydrates, which include glucose, fructose, cellobiose, trehalose, mannitol, sorbitol and derivatives of mucin [16–22]. Whilst carbohydrates are thought to be important in *C. difficile* colonisation of the gut and been reported to increase dysbiosis and mortality in a hamster model of CDI [12, 23] they are also reported to be protective against CDI. For example, high-carbohydrate diets containing microbiota accessible carbohydrates (MAC) showed faster clearance of *C. difficile* in mice due to the production of short-chained fatty acids (SCFA) [24], whilst a high-carbohydrate diet regardless of its composition protected against CDI [25]. The role of carbohydrates in CDI is therefore clearly complex, involving a number of interacting processes encompassing the host, its microbiota and *C. difficile *.

Trehalose is a disaccharide sugar consisting of two glucose molecules bonded by an α 1,1-glycosidic bond which in microorganisms can function as a nutrient source and protect against heat, cold or osmotic stress [26, 27]. Trehalose occurs naturally in foods such as mushrooms but is now widely used in a number of processed foods including ice cream and cereals as a sweetener and preservative [28, 29]. Trehalose can be metabolised by intestinal brush border trehalases as well as microbial-encoded trehalose-degrading enzymes [30, 31]. Gram-positive bacteria which can metabolise trehalose classically possess the *tre* operon, encoding genes for a trehalose-specific phosphotransferase system (PTS) transporter (*treP*), a phosphotrehalase (*treA*) for its subsequent breakdown into glucose and glucose-6-phosphate and a transcriptional regulatory protein (*treR*) which regulates its expression and senses trehalose [32–34].

The increased intake of trehalose into the diet was somewhat controversially proposed to have contributed towards the emergence of hypervirulence in *C. difficile *. This was evidenced by the ability of ribotype (RT) 027 and 017 strains to metabolise lower trehalose concentrations relative to non-hypervirulent strains *in vitro*, as a mutated TreR regulatory protein was thought to cause increased *treA* expression [18, 19]. Similarly, *in vitro*, RT078 strains can metabolise lower trehalose concentrations, except this occurs through increased uptake by a trehalose-specific PTS encoded by the *ptsT* gene [18]. Trehalose-fed mice was then shown to enhance the virulence of the hypervirulent RT027 R20291 during infection, with a RT027 strain able to induce the *treA* gene at a much higher level relative to mice infected with a non-hypervirulent strain [18]. However, this hypothesis has been disputed as these trehalose variants have been shown to be common within the *C. difficile* species and their presence were not associated with increased mortality and virulence [35, 36]. Further experimental models also showed that mice fed on trehalose and infected with a RT027 strain significantly reduces the abundance of *C. difficile* with no difference in mortality as compared to non-infected mice [37]. Finally, in an *in vitro* bioreactor model supplemented with trehalose, a RT027 strain was unable to stimulate CDI due to presumed increased competition by the remaining bacterial community, allowing only for spore germination and preventing toxin production [38]. It is thus clear that a narrative whereby trehalose metabolism contributes towards hypervirulence is not as straightforward as was once thought.

In order to further understand trehalose metabolism in *C. difficile* we utilised representative strains from each of the five toxigenic clades to investigate strain-specific differences that could occur during its utilisation. Our results suggest that the ability of the *C. difficile* species to metabolise trehalose is variable across strains as demonstrated by growth and gene expression analyses. Further, trehalose utilisation by hypervirulent *C. difficile* strains reduces toxin and sporulation gene expression levels. We demonstrate that the TreR regulatory protein can bind to the *treR* and *treA* promoter regions, as well as bind trehalose-6-phosphate increasing its binding ability to the *treA* promoter. Finally, we describe the induction of a putative trehalose metabolism pathway in the R20291 strain that has yet to be characterised in the *C. difficile* species. These results further characterise the molecular basis of *C. difficile* trehalose metabolism, thus providing a greater knowledge of the fundamental metabolic processes which aids in its colonisation of the gut.

## Methods

### Bacterial strains and culture conditions

*C. difficile* strains used in this study are listed in Table 1. *C. difficile* strains were routinely cultured under anaerobic conditions (N_2_/CO_2_/H_2_) in an anaerobic cabinet (Don Whitley Scientific, Shipley, UK) at 37°C. All strains were grown on fastidious anaerobe agar with horse blood (FAABL) (Oxoid, Basingstoke, UK) and allowed to incubate for 24 h. Unless otherwise stated, overnight cultures were carried out in brain heart infusion broth (37 g l^−1^) (BD Biosciences, New Jersey, USA) supplemented with 0.5 % (w/v) yeast extract (BD Biosciences, New Jersey, USA) (BHIS). TY medium (adjusted to pH 7.4 before autoclaving) [39] and 70 : 30 sporulation medium [40] were used for gene expression analysis of toxin and sporulation associated genes respectively.

**Table 1.** List of *C. difficile* strains used in this study

Strain	Relevant information	Source or reference
TL178 (Ribotype 002)	Representative strain – Clade 1	[68]
R20291 (Ribotype 027)	Representative strain – Clade 2	[90]
CD305 (Ribotype 023)	Representative strain – Clade 3	[68]
CF5 (Ribotype 017)	Representative strain – Clade 4	[68]
M120 (Ribotype 078)	Representative strain – Clade 5	[68]

### Whole genome sequencing and analysis

*C. difficile* TL178 and CD305 were grown for 48 h on Columbia blood agar (CBA) (Oxoid, Basingstoke, UK) supplemented with 5 % (v/v) defibrinated horse blood and a single colony was then used to inoculate 5 ml BHIS. After 48 h of growth, genomic DNA (gDNA) was isolated from each strain using the DNeasy UltraClean Microbial Kit (Qiagen, Manchester, UK) according to the manufacturer’s instructions. Library preparation was completed using the NEBNext Ultra II FS DNA Library Prep Kit, following the NEBNext Ultra II protocol for large fragment sizes (>550 bp) (New England BioLabs, Ipswich, UK) and subsequently sequenced on the Illumina MiSeq platform. Following 72 h sequencing run time, fastq files were quality checked using both FastQC (version 0.11.9) and MultiQC (version 1.9), trimmed with default parameters on fastp (version 0.20.0) [41–43]. *De novo* assembly was completed using SPAdes (version 3.13.1) [44]. To determine the quality of the assembled contigs, QUAST (v5.0.2) was used followed by MultiQC (v1.9) to combine all QUAST output files into a readable *html* for complete run analysis [42, 45].

### Bioinformatic analyses

To search for protein sequences of the trehalose metabolism proteins in selected *C. difficile* strains and other bacterial species, the R20291 (Genbank accession no. FN545816.1), CF5 (Genbank accession no. NC_017173.1), M120 (Genbank accession no. NZ_FRES01000002.1), *Bacillus subtilis* substr. *subtilis* str. 168 (Genbank accession no. CP010052.1), *Clostridium botulinum* ATCC 3502 (Genbank accession no. AM412317.1) and *Clostridium perfringens* str. 13 (Genbank accession no. BA000016.3) genomes were extracted from Basic Local Alignment Search Tool nucleotide (BLASTn), uploaded to the rapid annotation using subsystem technology (RAST) viewer [46] (http://rast.nmpdr.org) and annotated using the SEED database [47] to determine ORFs. Raw FASTA files of the sequenced TL178 and CD305 strains were uploaded in the same manner to annotate genomes. Protein sequences of trehalose metabolism genes from each strain were then aligned against one another using Clustal Omega alignment [48], and the percent amino acid identity was recorded. To identify putative TreR binding sites in within the *treR* and *treA* genes, the RegPrecise database [49] was used. To identify structural homology of selected proteins, the structural homology predictor Phyre2 [50] was used.

### Minimal medium growth assays

Defined minimal medium (DMM) was prepared as previously described [18] and supplemented with either 20 mM glucose, 10 mM trehalose or 50 mM trehalose as indicated. BHIS overnight cultures were adjusted to OD_600_ of approximately 0.05 in 5 ml DMM with the above carbon source and grown for 10 h in biological triplicate. Growth was monitored by measuring the OD_600_ every 2 h for 10 h with the readings averaged for each timepoint.

### RNA isolation and quantitative-reverse transcription PCR analysis (qRT-PCR)

To determine the expression levels of trehalose metabolism genes across the *C. difficile* representative strains, BHIS overnight cultures of each were sub-cultured to an OD_600_ of 0.05 in 25 ml of DMM supplemented with either 20 mM glucose, 10 mM trehalose or 20 mM glucose and 10 mM trehalose. These were grown to mid-logarithmic phase (strain dependent 4–6 h), mixed with an equal volume of ice-cold 1 : 1 ethanol: acetone mix and stored at −80 °C. For toxin gene expression analysis, the R20291 and M120 strains were grown in 20 ml TY medium with or without 10 mM trehalose until mid-logarithmic phase (OD_600_ 0.5) or T_3_ (3 h following OD_600_ 1.0) and harvested as above. For sporulation gene expression analysis, the R20291 and M120 strains were grown overnight in TY medium supplemented with 0.1 % taurocholate to induce germination of spores. Cultures were then back-diluted to OD_600_ 0.1 in 5 ml 70 : 30 medium, grown to OD_600_ 0.5 and then diluted 1 in 10 into 20 ml 70 : 30 medium with or without 10 mM trehalose and grown and harvested as described above for toxin gene expression analysis.

The cell suspension was then thawed on ice, centrifuged at 5000 ***g*** for 10 min at 4°C, supernatant decanted and the pellet resuspended in 5 ml 1 % beta-mercaptoethanol. The suspension was then centrifuged once more at 10000 ***g*** for 5 min at 4°C. RNA isolation was carried out using a RNeasy Mini Kit (Qiagen, Manchester, UK). Cells were resuspended in 200 µl TE buffer (10 mM Tris-HCl, 1 mM EDTA, pH 8.0) containing 15 mg ml^−1^ lysozyme and 1.5 mg ml^−1^ Proteinase K (Qiagen, Manchester, UK) and incubated for 10 min at room temperature followed by the addition of 700 µl buffer RLT with 1 % beta-mercaptoethanol. Cells were then transferred to a 2 ml Lysing Matrix B tube (MP Biomedicals, Cambridge, UK) and treated in a FastPrep-24 instrument (MP Biomedicals, Cambridge, UK) at a setting of 6.0 m s^−1^ for 45 s. The lysate was then centrifuged at 13000 ***g*** for 10 min and the supernatant added to 500 µl of 98 % ethanol before RNA isolation and clean-up using a RNeasy column by following the manufacturer’s instructions. The protocol was modified to include an initial on-column DNA digestion step using the RNase-free DNase set (Qiagen, Manchester, UK) according to the manufacturer’s instructions. RNA concentration and purity (260/280 and 260/230 ratios) were determined by a Nanodrop spectrophotometer and RNA was stored at −80 °C until needed.

DNA-free RNA was prepared using the TURBO DNA-free kit (Invitrogen, Renfrewshire, UK) following the manufacturer’s instructions, except for increasing the amount of TURBO DNase to 2 µl per sample. Following incubation at 37°C for 30 min, this was then repeated for a further 30 min at 37°C. Synthesis of cDNA was carried out using 0.5 µg RNA using the Superscript IV Vilo Mastermix (Invitrogen, Renewshire, UK) according to the manufacturer’s instructions. To confirm cDNA generation and removal of contaminating gDNA, PCR was carried out using *tpi* gene-specific primers (Table S1, available in the online version of this article) as compared to the no reverse transcriptase controls (RT-). cDNA was diluted 1 : 10 and 4 µl (10 ng) was used as template for qPCR using the PowerUp SYBR Green Master Mix (Applied Biosystems, UK) with a primer concentration of 0.5 µM on a LightCycler 480 II (Roche Diagnostics, UK). Each reaction was carried out in technical triplicate with each condition containing three biological replicates, with their corresponding RT- control to control for contaminating gDNA. qRT-PCR primers were designed using the PrimerQuest tool provided by Integrated DNA Technologies and primer efficiencies were determined by the generation of standard curves with PCR efficiencies between 90 and 110 %. Specificity of the reaction was determined by melting curve analysis as indicated by a single peak following thermocycling. Expression fold changes were calculated using the comparative cycle threshold method [51], with the expression of the amplified transcript normalised to that of the housekeeping gene, *rpoA*. Statistical significance between conditions were determined by one-way ANOVA with Tukey’s correction or unpaired t-test with or without Welch’s correction using GraphPad Prism nine as indicated.

### Reverse transcription-PCR (RT-PCR) and operon verification

DNA-free RNA was purified from *C. difficile* R20291 and M120 cultures grown in 10 mM trehalose and reverse transcribed into cDNA as described above. cDNA was used as a template for PCR using the primer sets described in Table S1 under ‘Reverse transcription-PCR’. For the respective assay, gDNA from *C. difficile* R20291 and M120 was used as a positive control and a RT- control was used to check for gDNA contamination. PCR products were separated by agarose gel electrophoresis on a 2 % agarose gel and visualised by SYBR Safe DNA gel stain (Invitrogen, Renfrewshire, UK).

### Electrophoretic mobility shift assay (EMSA)

Recombinant C-terminally His-tagged TreR was produced commercially by GenScript (Piscataway, NJ). DNA probes were designed to encompass the upstream regions of the *treA* and *treR* genes which included the putative TreR binding sites as described in the text. Biotinylated DNA probes were generated through PCR after amplification of *C. difficile* R20291 gDNA using Phusion Hot Start II DNA polymerase (Thermo Fisher Scientific, Massachusetts, USA) and 5′ biotin-labelled primers, as described in Table S1, followed by their purification using a QIAquick PCR Purification kit (Qiagen, Manchester, UK). Then 1 nM of biotinylated DNA was incubated with recombinant TreR protein (0–1.25 µM) in 1X EMSA binding buffer (10 mM Tris-HCl, pH 7.5, 50 mM KCl, 1 mM DTT, 50 ng µl^−1^ Poly (dI-dC), 10 % glycerol, 10 mM MgCl_2_, 0.05 % NP-40) for 30 min at 37°C. For experiments involving trehalose-6-phosphate as an effector, this was added to a final concentration of 10 mM and incubated as above. Samples were separated using electrophoresis in a 6 % 0.5X Tris-borate EDTA (TBE) polyacrylamide gel (Invitrogen, Renfrewshire, UK) for 2 h 30 min at 4°C, which was prerun at 100V for 30 min in 0.5X TBE buffer at 4 °C. Next, DNA was transferred by electroblotting to a positively charged Biodyne B nylon membrane (Thermo Fisher Scientific, Massachusetts, USA) at 100V for 30 min at 4°C which was then UV crosslinked at 302 nm for 15 min. The biotinylated probes were then detected using the Lightshift chemiluminescent EMSA kit (Thermo Fisher Scientific, Massachusetts, USA) according to the manufacturer’s instructions and imaged using a Syngene G box imager.

### Limited proteolysis assay

Trypsin digestion of the TreR protein (2.5 µM) was carried out in a 180 µl volume containing 25 mM Tris-HCl, pH 7.5, 10 mM CaCl_2_ and 1.5 ng µl^−1^ trypsin (Promega, Southampton, UK) in the presence or absence of 10 mM trehalose-6-phosphate or trehalose at 37°C. Aliquots of 20 µl volumes were withdrawn at indicated timepoints, quenched by the addition of 6.5 µl of 4X SDS-PAGE loading buffer, boiled at 95°C for 10 min and stored at −20°C overnight. Then 15 µl of sample was then separated by electrophoresis on a 4–20 % TGX precast polyacrylamide gel (Bio-Rad Laboratories, California, USA) followed by staining using the GelCode blue safe protein stain (Thermo Fisher Scientific, Massachusetts, USA).

### RNA-sequencing analysis

*C. difficile* R20291 was grown overnight in BHIS, diluted to OD_600_ 0.05 in DMM supplemented with 20 mM glucose or 10 mM trehalose and allowed to grow to mid-logarithmic phase (approximately 6 h). Cultures were then harvested and processed for RNA isolation and DNase treatment as above and stored at −80°C. RNA-seq and bioinformatic analysis was performed at Deepseq (University of Nottingham, UK).

RNA samples were shipped to Deepseq and upon receipt, concentrations were measured using the Qubit RNA BR Assay Kit (ThermoFisher Scientific, Massachusetts, USA). RNA integrity was assessed using the Agilent 4200 TapeStation and the Agilent RNA ScreenTape Assay (Agilent, SantaClara, USA) with RNA samples of RIN >8.0 being used for subsequent procedures. Ribodepletion was performed on 0.5 µg of total RNA, using the Qiagen FastSelect-5S/16S/23S ribodepletion kit (Qiagen, Manchester, UK) using the protocol: FastSelect-5S/16S/23S with NEBNext Ultra II Directional Library Prep Kit. Indexed sequencing libraries were then prepared using the NEBNext Ultra II Directional RNA Library Preparation Kit for Illumina (New England BioLabs, Ipswich, UK) and NEBNextMultiplex Oligos for Illumina (96 Unique Dual Index Primer Pairs) (New England Bio Labs, Ipswich, UK). Libraries were quantified using the Qubit Fluorometer and the Qubit dsDNA HS Kit (Thermo Fisher Scientific, Massachusetts, USA). Library fragment-length distributions were analysed using the Agilent TapeStation 4200 and the Agilent High Sensitivity D1000 ScreenTape Assay (Agilent, Santa Clara, USA). Libraries were pooled in equimolar amounts and final library quantification performed using the KAPA Library Quantification Kit for Illumina (Roche; Diagnostics, UK). The library pool was sequenced on the Illumina NextSeq 500 on a NextSeq500 High Output 300 cycle kit (Illumina, San Diego, USA), to generate over 10 million pairs of 150 bp paired-end reads per sample. Bioinformatics analysis was carried out by Deepseq as described below.

Differential gene expression analysis was carried out by mapping the sequenced reads to the *C. difficile* R20291 reference genome (https://www.ncbi.nlm.nih.gov/nuccore/FN545816.1). Reads were filtered by using the trimming pipeline, which filtered low sequencing score and reads aligned to adapter sequences. Raw reads were trimmed against adaptors and then reads were quality trimmed by skewer. Reads that passed the filter were then mapped onto the reference genome by the hisat2 mapping tool. Mapped reads were then counted by using the ‘featurecounts’ tool (http://subread.sourceforge.net/) to determine the number of uniquely and correctly aligned reads per gene. Normalised read counts (RPKM) for a given gene were determined by normalising the exon space of a gene against the total number of mapped reads (excluding rRNA and reads not uniquely mapped according to their mapping quality score [MAPQ] using MAPQ20) in an alignment file and against the total length of the gene’s exon space. Differentially expressed genes were then determined using the DESeq package, which analyses the variance between biological replicates within the RNA-seq analysis in order to better model the expression values of individual genes within the group of replicates. DESeq then determines differentially expressed genes for each comparison by using, *p* value= ≤0.05.

## Results

### Trehalose metabolism genes are conserved in *C. difficile *, but atypical in their arrangement relative to other bacterial species

**Fig. 1.**
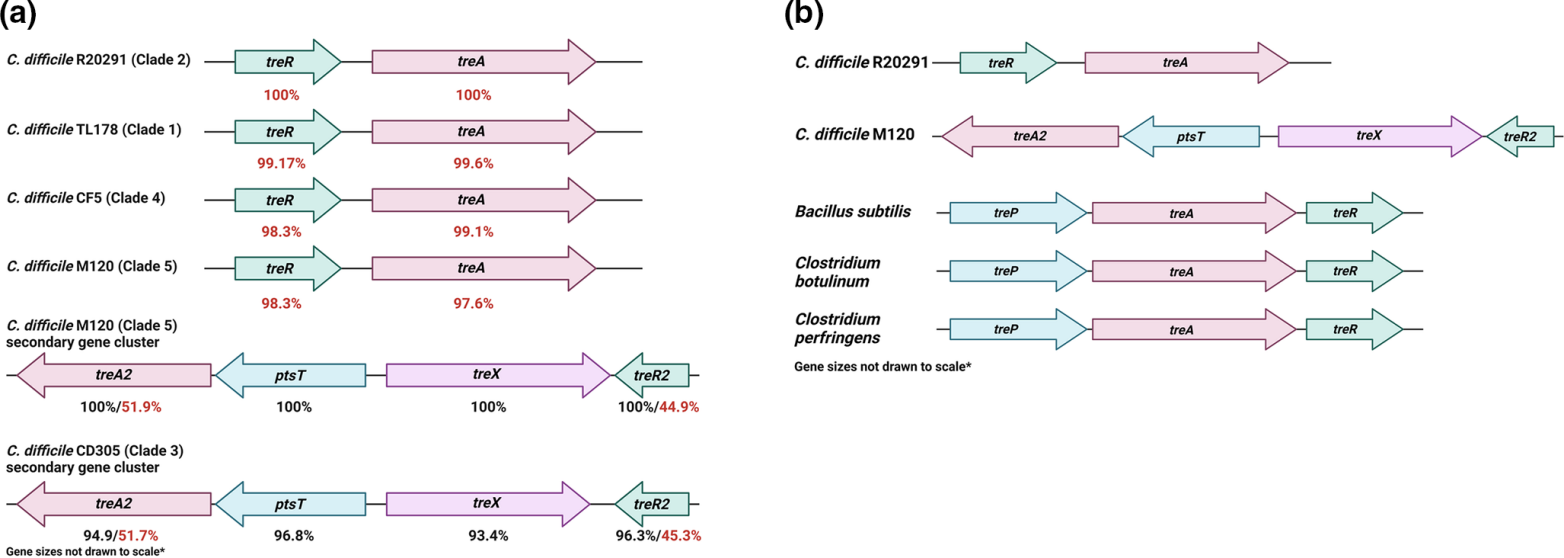
*
C. difficile* trehalose metabolism genes are variable across the species. (**a**) Genomic organisation of the trehalose metabolism genes in *C. difficile *. Numbers below arrows indicate percent amino acid identity as compared to *C. difficile* R20291 (*treR* and *treA*) in red text or *C. difficile* M120 (*ptsT*/*treP*) in black text. (**b**) Genomic organisation of the trehalose metabolism genes in *C. difficile* R20291 (*treR* and *treA*), M120 (*treA2*, *ptsT*, *treX* and *treR2*), *Bacillus subtilis* substr. subtilis str. 168, *Clostridium botulinum* ATCC 3502, *Clostridium perfringens* str. 13. Created with Biorender.com.

Analysis of genes and their protein products known to be associated with trehalose metabolism was carried out across the representative *C. difficile* strains used. *C. difficile* R20291 and M120 strains were used to anchor alignments for the TreR and TreA and the PtsT and TreX proteins respectively. *C. difficile* TL178, CF5 and M120 strains showed >97 and 98 % amino acid identity respectively towards TreR and TreA, whereas for M120 and CD305 strains, their TreR2 and TreA2 showed only ~45 % and ~52 % amino acid identity respectively (Fig. 1a). Interestingly when comparing between TreR2 and TreA2 from the CD305 and M120 strains amino acid identities were 96.3 and 94.9% respectively (Fig. 1a). Finally, the amino acid identities were ~96 % and ~93 % for PtsT and TreX respectively when the CD305 strain was compared to M120 strain, however, of note was that the TreX protein in CD305 was truncated by 103 amino acids (Fig. 1a).

*C. difficile* trehalose metabolism genes were then found to have an atypical arrangement when compared to other pathogenic *Clostridium* species and the model endospore-forming organism, *Bacillus subtilis *. Specifically the *ptsT* gene (*treP* in *B. subtilis *) is not found in proximity to the *treR* and *treA* genes with no trehalose-specific PTS genes annotated as of yet in any strains except those with the secondary gene cluster (SGC) (Fig. 1b). Further, the SGC is not arranged in the same manner as other trehalose metabolism gene loci in *B. subtilis* and other *Clostridium* species, most notably the presence of the *treX* gene encoding a putative trehalase (Fig. 1b).

### Trehalose utilisation is variable across *C. difficile*

**Fig. 2.**
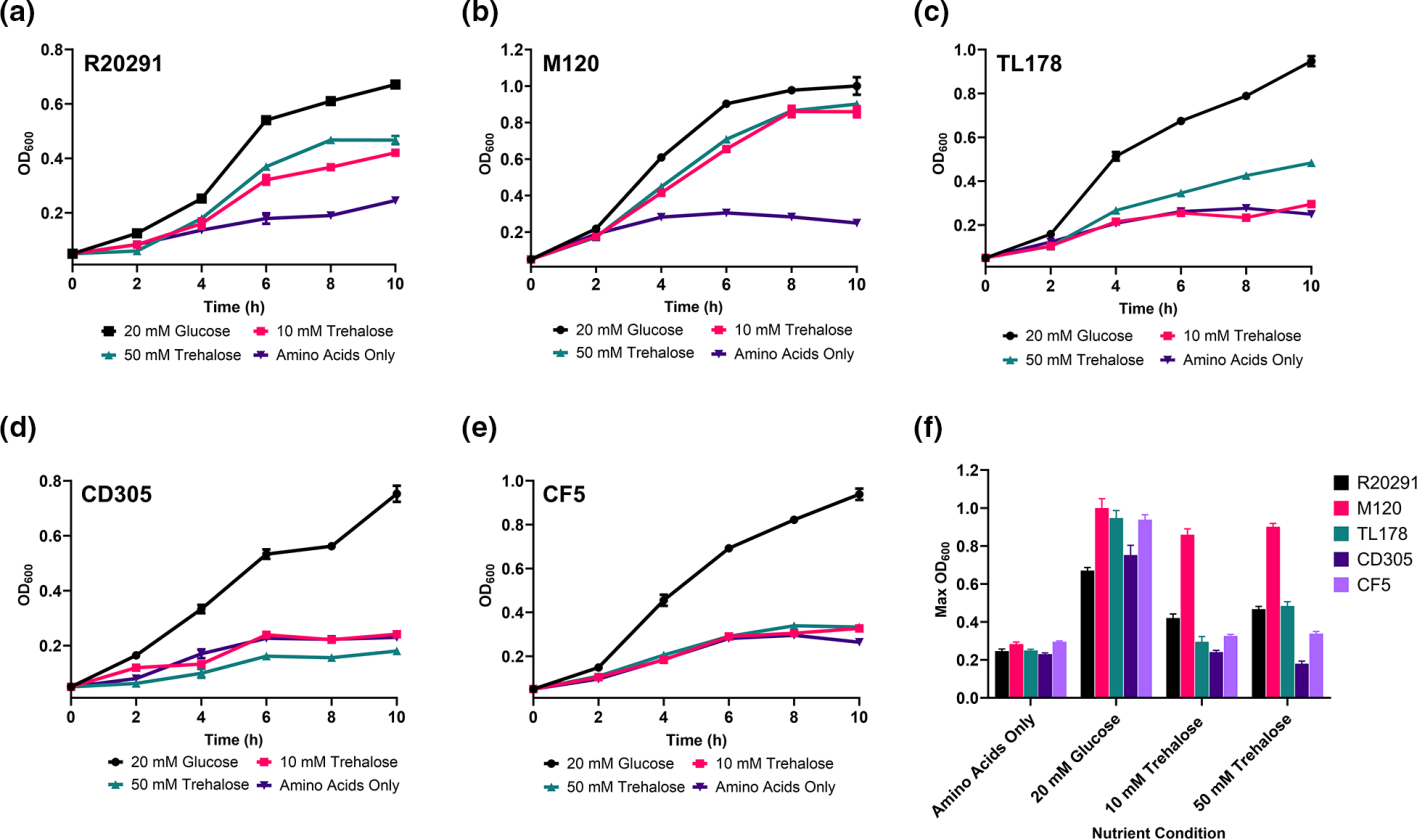
Representative *C. difficile* strains show varying levels of growth in trehalose. Growth curve of *C. difficile* (**a**) R20291, (**b**) M120, (**c**) TL178, (**d**) CD305 and (**e**) CF5 strains in DMM supplemented with 20 mM glucose, 10 mM trehalose, 50 mM trehalose or amino acids only. (**f**) Maximum cell density (OD_600_) of *C. difficile* R20291, M120, TL178, CD305 and CF5 cultures in different nutrient conditions. Data represents three biological replicates with error bars representing standard error of the mean.

Given the diversity in nature and arrangements of the trehalose metabolism genes across the *C. difficile* strains of interest we wanted to see what impact this had upon their ability to utilise trehalose as the sole carbon source. Only the hypervirulent R20291 and M120 strains displayed increase growth in 10 mM (low) trehalose and 50 mM (high) trehalose (Fig. 2a, b, f). The TL178, CD305 and CF5 strains showed only marginal increases in growth in 10 mM trehalose, whereas in 50 mM trehalose, the TL178 strain was capable of increased growth which was greater than that of the CD305 and CF5 strains (Fig. 2c, e, f).

### Expression levels of *C. difficile* trehalose metabolism genes vary across representative strains, but in some cases do not correlate to increased growth

In order to understand more about the transcriptional regulation of trehalose metabolism, we used RT-PCR to determine if *treR* and *treA* are co-transcribed. Using cDNA derived from the *C. difficile* R20291 strain grown in trehalose, PCR products could be amplified across the adjacent ORFs for *treR* and *treA* with no amplification detected in the no reverse transcriptase controls (Fig. S1A), demonstrating their co-transcription. Similarly, we determined that *ptsT* and *treA2* genes are co-transcribed in *C. difficile* M120 strain during growth in trehalose (Fig. S1B).

**Fig. 3.**
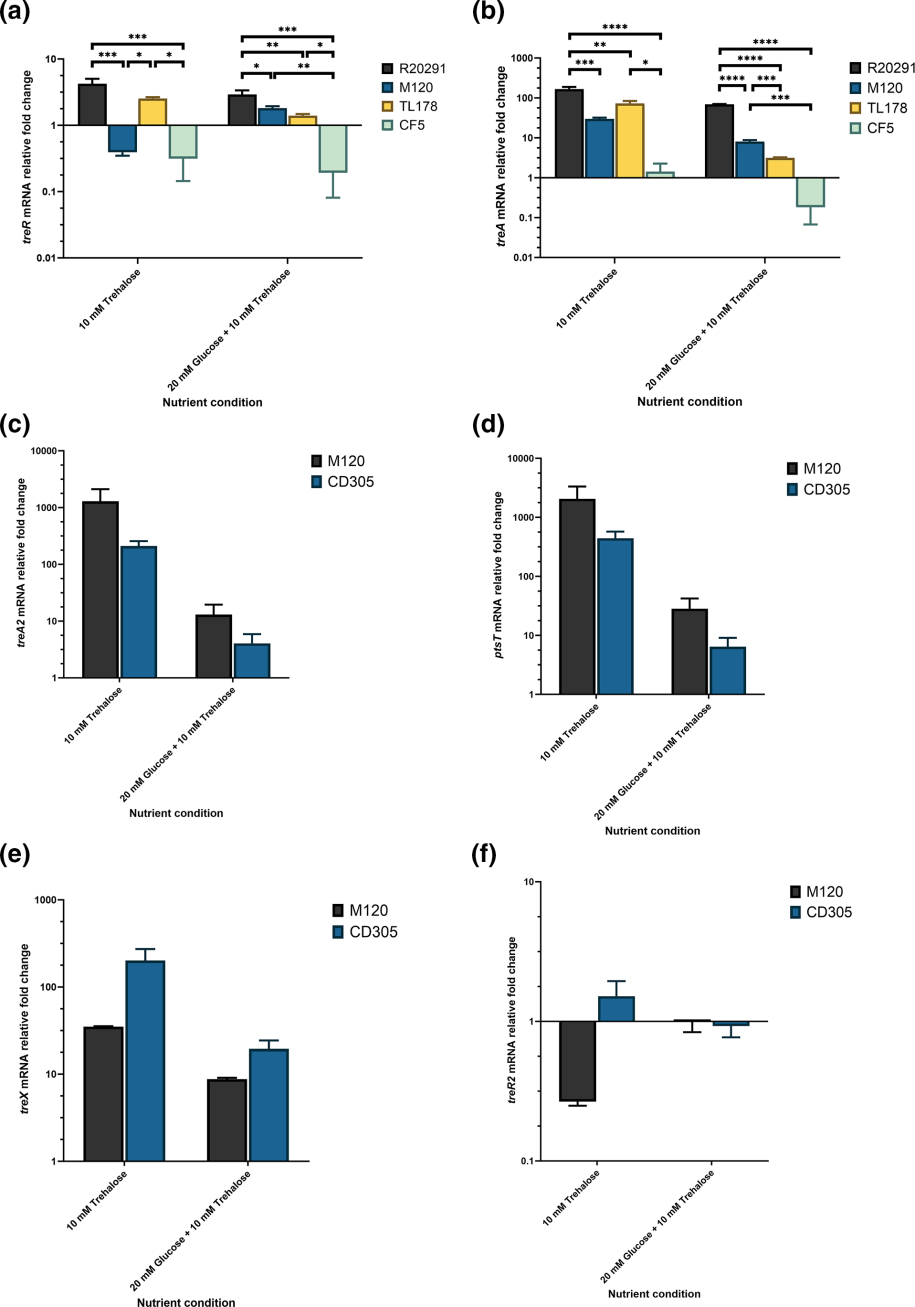
*
C. difficile* trehalose metabolism genes are responsive to trehalose across the five representative strains. Cultures of *C. difficile* R20291, M120, TL178, CD305 and CF5 were grown in DMM supplemented with 10 mM trehalose or 20 mM glucose and 10 mM trehalose to mid-logarithmic phase, harvested for RNA extraction and processed for cDNA generation and qRT-PCR analysis as described in Methods. mRNA relative fold change of the (**a**) *treR*, (**b**) *treA*, (**c**) *treA2*, (**d**) *ptsT*, (**e**) *treX* and (**f**) *treR2* genes are relative to that of the respective strain grown in DMM supplemented with 20 mM glucose. Data represents three biological replicates showing standard error of the mean. Statistical significance of the *treR* or *treA* gene expression levels compared between strains for each nutrient condition was completed using a one-way ANOVA with Tukey’s correction. Similarly, statistical significance of the *treA2*, *ptsT*, *treX* and *treR2* genes between strains for each nutrient condition was completed using an unpaired t-test. Adjusted *p* values represented as *, *P*≤0.05, **, *P*≤0.01, ***, *P*≤0.001, ****, *P*≤0.0001.

Given the variance in observed growth phenotypes when trehalose was present as the sole carbon source we wanted to determine differences, if any, in the transcriptional level of trehalose metabolism genes. Relative to glucose-grown conditions, the *treR* and *treA* genes were induced in all *C. difficile* strains, with the exception of the *treR* gene in M120 and CF5 which was repressed (Fig. 3a, b). Although the *treR* and *treA* genes were induced across the majority of strains investigated, the levels varied between each with the R20291 strain showing the highest relative increase in transcription levels with a four-fold and 167-fold increase in transcript abundance respectively (Fig. 3a, b). Given the major role of carbon catabolite repression in regulating carbohydrate metabolism during glucose-grown conditions [52], we then determined if the trehalose metabolism gene expression levels were repressed when *C. difficile* strains were grown in glucose and trehalose-containing medium. Consistently, *treR* and *treA* gene transcript abundance were lowered when representative strains were cultured in 20 mM glucose and 10 mM trehalose relative to 10 mM trehalose, with the exception of the M120 *treR* gene which increased expression, displaying partial levels of induction suggesting incomplete carbon catabolite repression (Fig. 3a, b).

The SGC *treA2*, *ptsT*, *treX* and *treR2* genes were induced in the M120 and CD305 strains, except for the M120 *treR2* gene which was repressed (Fig. 3c–f). Similarly as above, growth of the M120 and CD305 strains in glucose and trehalose decreased the transcript abundance of each gene leading to partial induction, with the exception of the M120 *treR2* gene which increased (Fig. 3c, f). The M120 exhibited the highest level of induction of the *treA2* and *ptsT* genes, whilst the CD305 strain showed the highest induction in the *treX* gene in transcript abundance when they were cultured in trehalose (Fig. 3c–f). As the TL178, CD305 and CF5 strain were unable to attain growth when cultured in 10 mM trehalose even though their trehalose metabolism genes were induced, this suggests that trehalose utilisation by *C. difficile* is heterogenous and highly variable between individual strains.

### Trehalose lowers the expression of toxin and sporulation associated genes in hypervirulent *C. difficile* strains

**Fig. 4.**
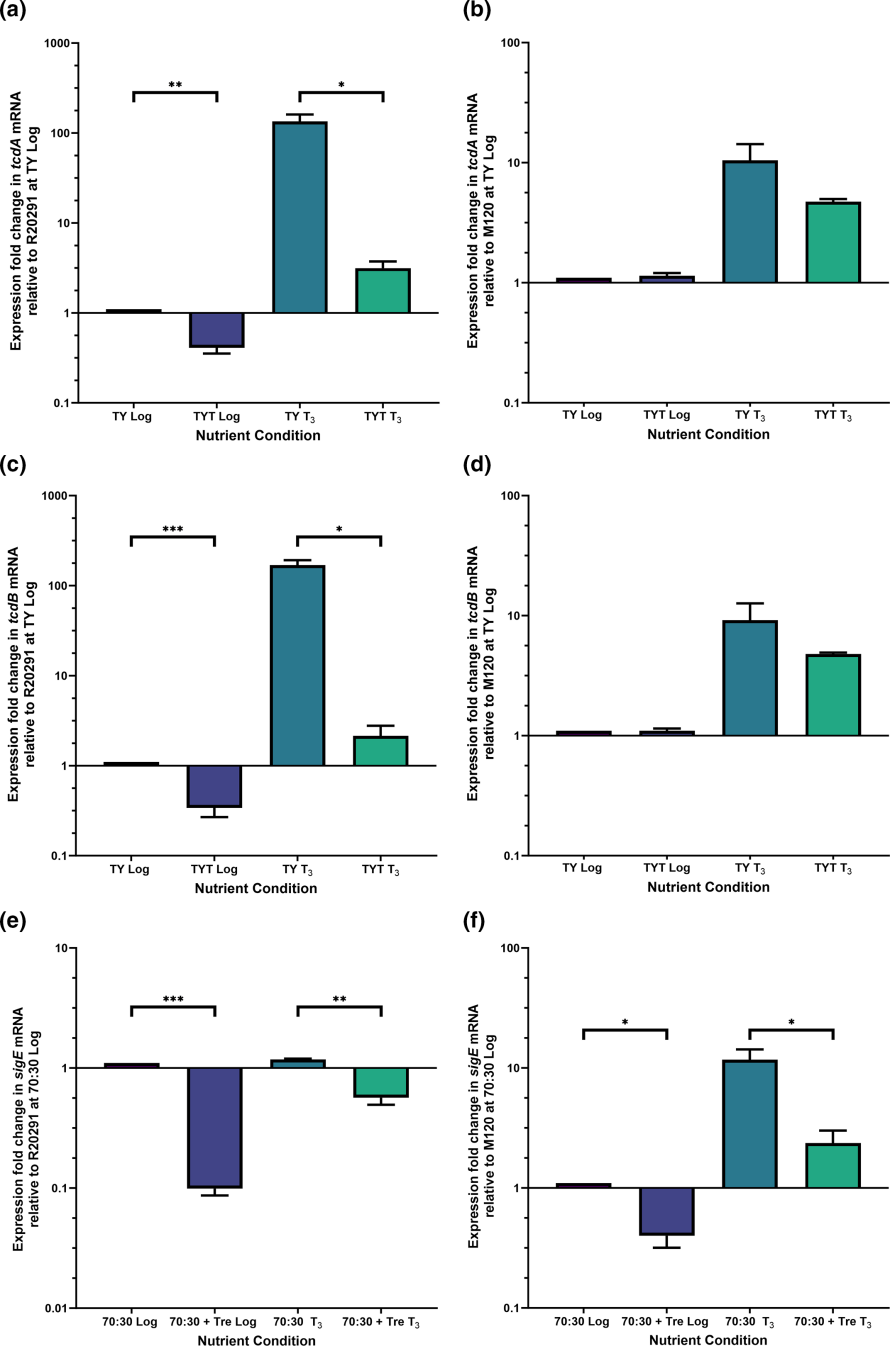
Trehalose reduces the expression of toxin and sporulation-associated genes in hypervirulent *C. difficile* strains *in vitro*. Cultures of *C. difficile* R20291 and M120 were grown in either TY or 70 : 30 medium with or without 10 mM trehalose (TYT and 70 : 30+Tre respectively) to mid-logarithmic phase (Log) and 3 h following the transition into stationary phase (**t_3_**), harvested for RNA extraction and processed for cDNA generation and qRT-PCR analysis as described in Methods. mRNA relative fold change of the *tcdA* (**a and b**), *tcdB* (**c and d**) and *sigE* (**e and f**) genes in the R20291 (**a, c and e**) and M120 strains (**b, d and f**) are relative to that of the respective *C. difficile* strain grown in TY (*tcdA* and *tcdB* genes) or 70 : 30 (*sigE* gene) Log. Data represents three biological replicates and error bars showing standard error of the mean. Statistical significance was compared at each timepoint (Log or T_3_) and condition using the unpaired *t*-test with Welch’s correction. Adjusted *p* values represented as *, *P*≤0.05, **, *P*≤0.01, ***, *P*≤0.001.

Given the proposed involvement of trehalose in the emergence of hypervirulence in *C. difficile* [18], we investigated the effect of trehalose on genes associated with pathogenesis in the hypervirulent R20291 and M120 strains. Expression of *tcdA* and *tcdB* were significantly reduced when the R20291 strain was grown in trehalose (Fig. 4a, c) but were not significantly decreased in M120 (Fig. 4b, d). Similarly, the *sigE* early sporulation sigma factor gene expression was significantly reduced for both the R20291 and M120 strains when grown in trehalose (Fig. 4e, f).

### The TreR transcriptional regulator binds to the promoter regions of the *treR* and *treA* genes and may have an activator function

*treR* encodes a protein annotated as a GntR-family transcriptional regulator (*CDR20291_2929*). Using InterProScan [53], the structure of TreR showed the presence of a conserved N-terminal winged-helix-turn-helix DNA-binding domain and a C-terminal effector-binding domain homologous to the UbiC transcription regulator-associated (UTRA) domain. The latter domain is characteristic of regulatory proteins which belong to the HutC subfamily of GntR regulators, which recognise a consensus palindromic DNA sequence represented by 5′GT(NTAN)AC-3′ [54]. Using the RegPrecise database, one sequence within the *treR* promoter region showed homology to the HutC consensus sequence, whilst the *treA* promoter region showed two sequences (Fig. S2A), with each sequence found to be conserved in the representative strains which contain the *treR* and *treA* genes (Fig. S2B).

**Fig. 5.**
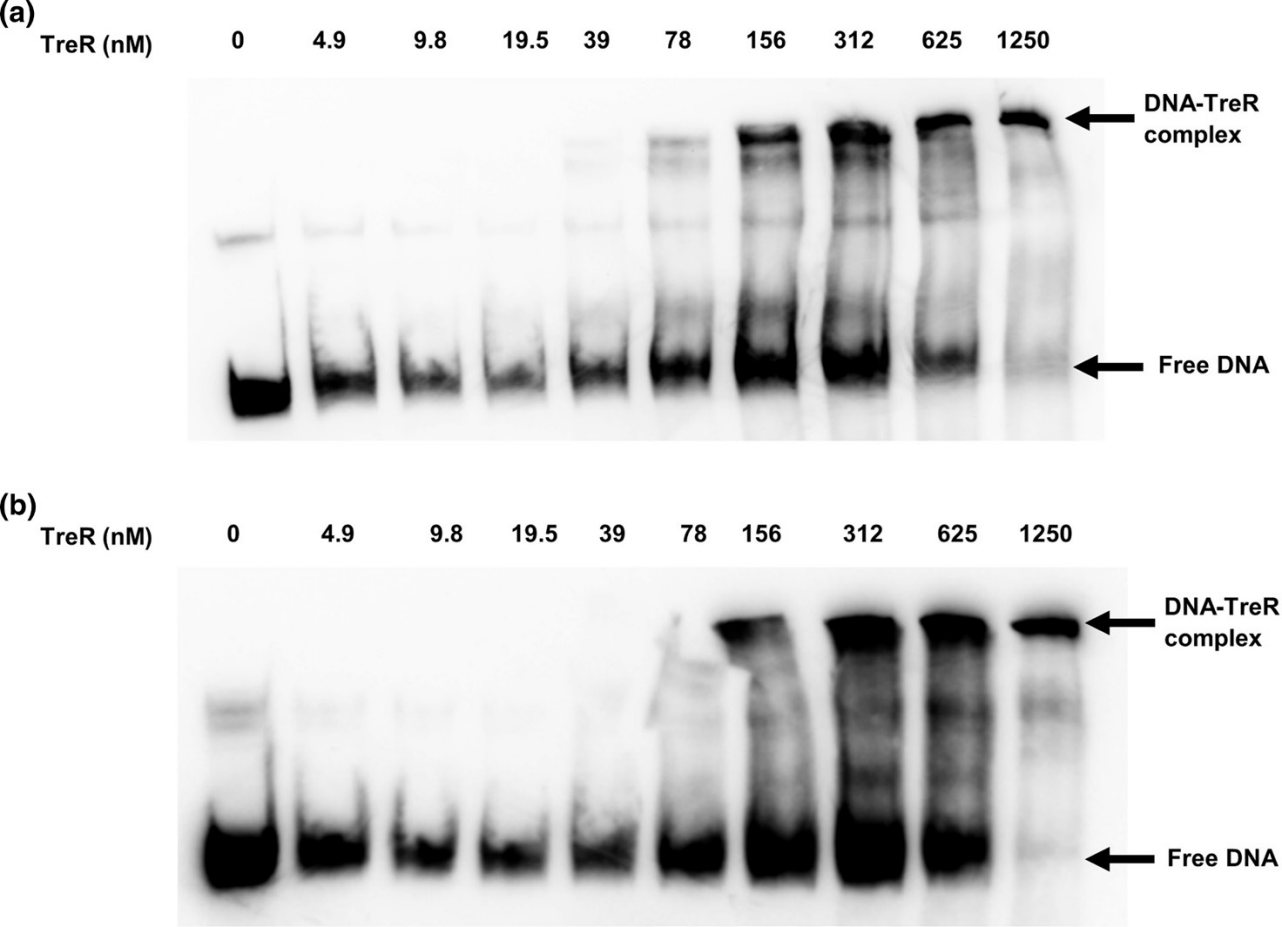
TreR can bind the promoter region of the *treR* and *treA* genes. Electrophoretic mobility shift assays (EMSA) were carried out by incubating increasing amounts of the TreR protein with biotinylated DNA probe (1 nM) of either the (**a**) *treA* or (**b**) *treR* promoter regions at 37 °C for 30 min. Samples were resolved on a 6 % 0.5X TBE polyacrylamide gel and visualised using chemiluminescence detection, 0 nM TreR represents negative control of no binding activity.

Electrophoretic mobility shift assays (EMSA) were used to determine if TreR could bind to the putative binding sites identified through bioinformatic analysis. When the TreR protein from R20291 was recombinantly produced and incubated with biotinylated DNA probes encompassing the putative TreR binding sites this resulted in visible DNA-protein complexes, thus validating TreR DNA-binding activity towards the *treR* and *treA* promoter regions (Fig. 5a, b).

**Fig. 6.**
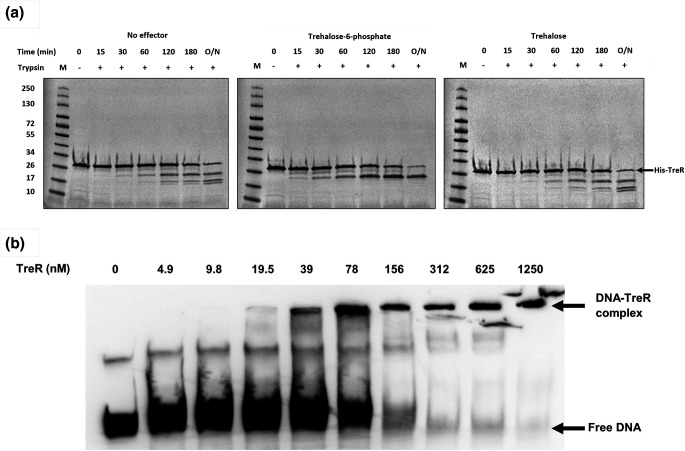
Trehalose-6-phosphate specifically interacts with TreR *in vitro*. (**a**) SDS-PAGE gels show the trypsin digest pattern of TreR incubated without effector (left panel), 10 mM trehalose-6-phosphate (middle panel) and 10 mM trehalose (right panel). M indicates molecular weight ladder (kDa) and O/N indicates overnight incubation. (**b**) Electrophoretic mobility shift assays (EMSA) were carried out by incubating increasing amounts of the TreR protein in the presence of 10 mM trehalose-6-phosphate with biotinylated DNA probe (1 nM) of the *treA* promoter region at 37°C for 30 min. Samples were resolved on a 6 % 0.5X TBE polyacrylamide gel and visualised using chemiluminescence detection, 0 nM TreR represents negative control of no binding activity.

Additionally, within the HutC subfamily of GntR regulators the C-terminal effector-binding domains recognise and bind effector molecules resulting in a conformational change altering their DNA-binding activity [55]. To ascertain if TreR possesses this effector binding activity, limited proteolysis assays were firstly carried out to determine the cognate effector it specifically binds. Compared to TreR alone and in the presence of trehalose, incubation with trehalose-6-phosphate led to an altered TreR trypsin digest pattern (Fig. 6a). This suggests that trehalose-6-phosphate is the specific effector of TreR as it protects against trypsin digestion due to a conformational change upon its interaction. Next to determine the effect of trehalose-6-phosphate on DNA-binding activity, TreR was incubated with biotinylated *treA* DNA probe in the presence of trehalose-6-phosphate and subject to EMSA analysis. Trehalose-6-phosphate increased the shift of DNA-protein complexes (Fig. 6b), suggesting this interaction may stabilise the TreR-DNA complex *in vitro* by positively influencing its binding ability with the *treA* promoter DNA probe.

### RNA-sequencing analysis revealed a putative trehalose metabolism pathway in the *C. difficile* R20291 strain

**Fig. 7.**
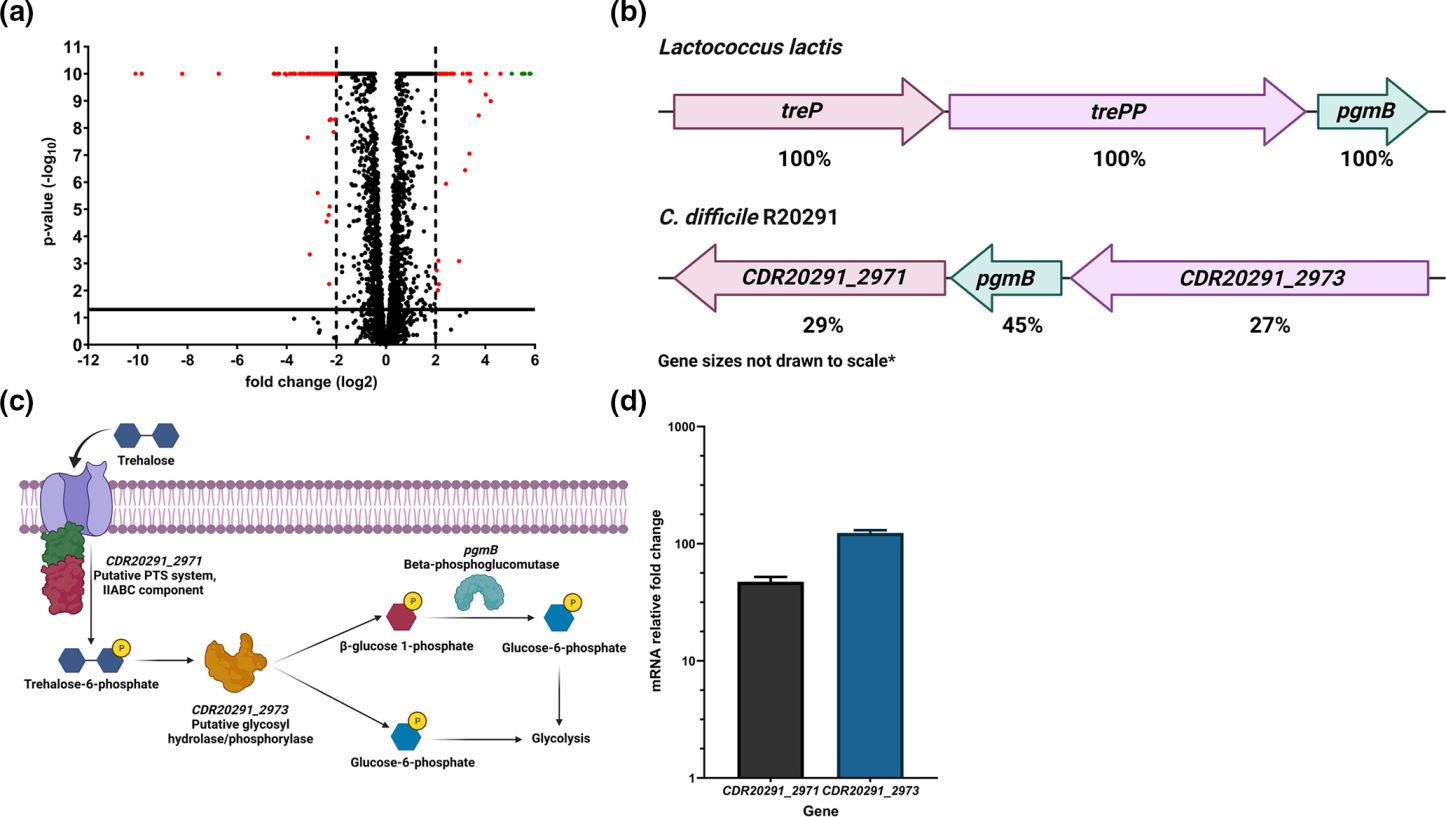
RNA-seq reveals an unidentified putative trehalose metabolism pathway. (**a**) Volcano plot showing RNA-seq analysis comparing gene expression changes of *C. difficile* R20291 grown in trehalose to growth in glucose. Vertical dashed lines represents genes (red dots) with a fold change above (≥ 2) or below (≤ −2) the threshold cut-off. Horizontal black line represents differentially expressed genes with a *p* value ≤0.05. Samples with a *p* value >1 x 10^−10^ are represented as 10 on the plot. Green dots represent the highest upregulated genes in the dataset consisting of the putative trehalose metabolism genes, *CDR20291_2971*, *pgmB*, *CDR20291_2973* and *ascB3* and the *treA* gene. (**b**) Genomic organisation of the *Lactococcus lactis* trehalose metabolism genes and *C. difficile* R20291 putative trehalose metabolism genes. *treP*=trehalose-specific PTS, *trePP*=trehalose-6-phosphate phosphorylase, *pgmB*=beta-phosphoglucomutase. Numbers below arrows indicate percent amino acid identity as compared to *Lactococcus lactis *. Created with Biorender.com. (**c**) Proposed metabolic pathway of the upregulated putative trehalose metabolism genes in *C. difficile* R20291. Trehalose is transported into the cell through a putative PTS system IIABC component (*CDR20291_2971*) which is concomitantly phosphorylated to trehalose-6-phosphate. Trehalose-6-phosphate is then cleaved by a putative glycosyl hydrolase/phosphorylase (*CDR20291_2973*) into glucose-6-phosphate and β-glucose 1-phosphate. β-glucose 1-phosphate is then converted to glucose-6-phosphate by a beta-phosphoglucomutase (*pgmB*). The produced glucose-6-phosphate molecules then enter glycolysis. Metabolic pathway adapted from Andersson *et al*., [56], using *Lactococcus lactis* as the model organism. Created with Biorender.com. (**d**) Cultures of *C. difficile* R20291 were grown in DMM supplemented with 10 mM trehalose to mid-logarithmic phase, harvested for RNA extraction and processed for cDNA generation and qRT-PCR analysis as described in Methods. mRNA relative fold change of the *CDR20291_2971* and *CDR20291_2973* genes are relative to that of growth in DMM supplemented with 20 mM glucose. Data represents three biological replicates showing standard error of the mean.

To determine, for the first time, the effect of trehalose utilisation on the transcriptome of *C. difficile *, RNA-sequencing (RNA-seq) was performed on the hypervirulent R20291 strain comparing growth in trehalose to that in glucose nutrient conditions. Forty upregulated and 81 downregulated genes with a fold-change of ≥2 or ≤ −2 with a *p* value of ≤0.05 were identified (Fig. 7a and Table S2). Overall many of these changes could be related to the relief of carbon catabolite repression within *C. difficile* [52]. Amongst these, *treA* and four genes located within the same genetic locus (*CDR20291_2971*, *pgmB*, *CDR20291_2973*, and *ascB*) showed the highest upregulated fold-change (5.07 to 5.82 log_2_ change).

The induction of the *CDR20291_2971*, *pgmB* and *CDR20291_2973* genes were of particular interest as these are annotated as a putative PTS system IIABC component, a beta-phosphoglucomutase and a putative glycosyl hydrolase respectively. This gene arrangement is similar to a genetic locus in *Lactococcus lactis* which consists of a trehalose-specific PTS, a trehalose-6-phosphate phosphorylase and a beta-phosphoglucomutase which is essential for trehalose metabolism (Fig. 7b, c) [56]. qRT-PCR validation corroborated the RNA-seq results, showing the upregulation of the *CDR20291_2971* and *CDR20291_2973* genes during culture of R20291 in trehalose as compared to glucose grown conditions (Fig. 7d). Comparison of *CDR20291_2971* to the *C. difficile* M120 *ptsT* and *L. lactis* trehalose-specific PTS genes both showed 29 % identity in their amino acid sequences, suggesting putative functionality for trehalose import. We then endeavoured to identify the function of the putative *CDR20291_2973* gene using the structural homology predictor Phyre2, which showed structural homology to a maltose phosphorylase (27 % identity, 100 % confidence). Further, in the *C. difficile* 630 strain, this gene is annotated as a glycosyl hydrolase/phosphorylase, with InterProScan and BLASTp bioinformatic analysis identifying domains which facilitate phosphorylase activity as well as showing homology to number of other phosphorylase genes. Interestingly, these three genes are not present in all *C. difficile* strains across the five clades used in this study and found only in clades one, two and four, with each of these known to be lacking a known trehalose-specific PTS for trehalose transport.

## Discussion

Trehalose has been implicated in the emergence of hypervirulence in select *C. difficile* strains due to its more efficient utilisation *in vitro* [18, 19], however this has been disputed by several studies [35, 36, 38]. Additionally, gene expression analyses of *in vivo* CDI infection models have shown *treA* expression to increase, suggesting trehalose metabolism may be important during infection [14, 57]. Initial studies in defining *C. difficile* carbohydrate metabolism showed its ability to ferment trehalose amongst a panel of other carbohydrates, however the molecular basis of its metabolism is still poorly defined [22]. There is a large gap in our understanding of how different *C. difficile* strains respond to the same nutrient source, where to date only ethanolamine metabolism has been described [58]. Furthermore, *C. difficile* possesses a highly plastic genome and metabolic adaptations are thought to increase the chances of a strain causing recurrent infections [59]. This work aimed to further characterise *C. difficile* trehalose metabolism by combining bioinformatic, phenotypic, molecular and -omic techniques to provide a basis for understanding the consumption of this carbohydrate.

The representative strains used in this work possess identical trehalose metabolism genes as described by Shaw *et al*., [60], and here we show that a high level of conservation exists between these genes in respective strains suggesting they play an important role in *C. difficile* metabolism. Comparing these genetic loci to the *tre* operon in other bacterial species also highlighted a different genetic arrangement (Fig. 1b), therefore given these inter-strain and inter-species differences we defined growth profiles of each representative *C. difficile* strain in trehalose. We observed increased growth of the hypervirulent RT027 R20291 and RT078 M120 strains in 10 mM and 50 mM trehalose, which was consistent with previous accounts of trehalose utilisation by these strains, whereas the non-hypervirulent RT002 TL178, RT023 CD305 and RT017 CF5 strains did not [18, 19, 61]. Microevolution of *C. difficile* trehalose metabolism has been observed in the 630 strain from different labs, where each showed differing growth profiles during culture in 10 mM trehalose [62]. Starch and sucrose metabolism gene sequences have been shown to possess a high degree of variation across *C. difficile* strains, thought to occur due to their active evolution within a nutrient niche [62]. This may then provide the potential for metabolic adaptation towards trehalose utilisation over time in those strains which cannot metabolise it currently. For the metabolism of other nutrient sources, similar inter-strain growth differences have been observed during the growth of *C. difficile* 630 and VPI10643 in sorbitol which exhibit increased growth on this substrate relative to several other strains [63]. Further, *C. difficile* 630Δ*erm* has been shown to exhibit slightly increased growth compared to the R20291 strain when cultured in ethanolamine [58].

To delineate the observed growth variations between strains, qRT-PCR analyses for each gene were then carried out during growth in trehalose which highlighted a range of gene expression changes. Amongst the strains possessing the *treA* gene, the RT027 R20291 strain showed the highest level of induction, which was similar to expression levels in the RT027 CD2015 strain when compared to other non-RT027 strains [18, 19]. The entirety of the SGC in the RT078 M120 strain was then shown to be induced during its growth in trehalose, suggesting these genes may be involved in permitting this large growth increase relative to other tested strains. This highlights for the first time the functionality and responsiveness of these genes, however their specific roles in trehalose metabolism remain unknown. The trehalose metabolism genes which displayed partial induction in the presence of glucose also suggests that trehalose metabolism gene expression can overcome carbon catabolite repression. Interestingly, the increase in gene expression levels of the *treA*, *ptsT*, *treA2* and *treX* genes in the respective non-hypervirulent strains did not correlate to increased growth *in vitro*. This poses the question of which trehalose metabolism genes are essential across the strains to allow increased growth. In some cases, longer incubation times are required for *C. difficile* to achieve increased growth during *in vitro* growth on a specific carbohydrate. This was observed to permit increased growth on sorbitol, N-acetylglucosamine and mannose, therefore a prolonged incubation time may allow increased cell densities in these strains during growth in trehalose [16, 21, 63]. The genetic plasticity of *C. difficile* supports a great deal of phenotypic variation between strains affecting toxin production, sporulation and motility [64–67]. Metabolic variation between strains likely follows the same trend, as previously the clade four RT017 M68 strain was shown to display increased growth and expression of the *treA* gene in 10 mM trehalose, whereas our in work, the clade four RT017 CF5 strain could not [19]. These differences may have derived from the historic and recently emerged nature of the CF5 and M68 strains respectively, of which the latter has been shown to possess several discrete genomic regions [68]. These genetic differences may then serve to enhance the ability of the M68 strain to metabolise low concentrations of trehalose. These data then suggest a diverse metabolic response of trehalose metabolism in the *C. difficile* species, as even though respective genes are conserved across multiple strains, a great level of variability is apparent.

Nutrient availability is intimately associated with toxin production in *C. difficile* as this is thought to replenish nutrients to sustain growth within the gut environment [69]. Nutrients such as glucose and amino acids are sensed by the metabolic regulators CcpA and CodY respectively which repress virulence processes, when upon their limitation induces toxin gene expression [70, 71]. Prior *in vivo* work of mice infected with the R20291 strain indicated that trehalose consumption increased TcdB loads [18], therefore toxin and sporulation associated gene expression levels were assessed in the hypervirulent R20291 and M120 strains during *in vitro* growth in trehalose. Trehalose utilisation decreased *tcdA*, *tcdB* and *sigE* transcripts in both strains, an effect similar to how other nutrient sources regulate the expression of these *C. difficile* genes [16, 17, 39, 72]. Given that the breakdown product of trehalose-6-phosphate are glucose and glucose-6-phosphate, these metabolites are likely sensed by the CcpA regulator which then repress *tcdA*, *tcdB* and *spo0A* expression (as *spo0A* induction is required for *sigE* activation) [52, 70, 73].

The RT027 R20291 and RT078 M120 strains showed strain-specific differences in toxin and sporulation gene expression levels when utilising trehalose, even though growth conditions were identical for both (Fig. 4a–f). For example, during growth of the R20291 strain at stationary phase with no trehalose present, *sigE* expression was comparatively lower than that of the M120 strain under the same conditions (Fig. 4e, f). This may reflect known differences in sporulation efficiencies between these strains [67]. Other examples of strain-specific differences in toxin production and sporulation are well documented within *C. difficile* with a mutation in *tcdR*, a regulator of toxin gene expression, shown to decrease the amount of sporulation in the R20291 strain but conversely increase it in the 630Δ*erm* strain [74]. Similarly, mutation of the master sporulation regulator gene, *spo0A*, did not affect the amount of toxin produced in the 630Δ*erm* strain whilst in similar mutant strains of R20291 and RT078 it increased [75, 76]. Further a mutation in the *sigL* alternative sigma factor gene increased toxin production in the R20291 strain whereas a decrease was observed in a RT078 strain [77]. Finally, the same group showed the amount of toxin produced in wild-type R20291 to be higher than that of a wild-type RT078 strain, whilst the latter produced more spores than the former [77]. Further work investigating the trehalose-dependent effects on toxin and sporulation expression changes with other *C. difficile* strains will be useful in illuminating if these virulence processes are consistently reduced. To note, a limitation of this current work was the lack of assays which measure functional toxin levels and sporulation frequency. Importantly, although our current findings could be viewed as conflicting with the proposal that trehalose increases *C. difficile* virulence [18], our work was carried out under defined pure culture conditions *in vitro* which does not replicate the complex microbe-host and microbe-microbe interactions within the gut. In the future more physiologically-relevant growth conditions which recapitulate the gut microenvironment, e.g. in faecal water [78] or mouse caecal filtrate [79], could be used *in vitro* to investigate how trehalose utilisation impacts *C. difficile* virulence further. Nonetheless, our findings further perpetuate the controversial role of trehalose in *C. difficile* pathogenesis.

Regulation of trehalose metabolism in *C. difficile* is ill-defined, therefore we investigated the possibility of operonic structures between adjacent genes which showed that *treR* and *treA* and *ptsT* and *treA2* are co-transcribed (Fig. S1). Interestingly, the *treR-treA* intergenic region is unusually long (197 bp), where *in silico* analyses have shown the *treA* gene to possess a −35 and −10 consensus sequence in its upstream region [80]. Additionally, the *treR* upstream region lacks the −35 and −10 consensus sequence, instead possessing an extended −10 (TGn) motif, which may explain the lesser amounts of gene expression levels relative to the *treA* gene across the *C. difficile* strains used [80, 81]. Similar observations have been found for the *glpEGR* operon in *Escherichia coli *, where individual genes display differential rates of transcription as each possess their own promoter [82]. Unexpectedly, we observed TreR binding activity to both the *treR* and *treA* gene promoter regions even though these are co-transcribed, suggesting these genes may be differentially expressed by its regulatory control. Similar to this, the *grlRA* operon in *Citrobacter rodentium* was shown to be co-transcribed even though these two genes possess their own promoters, where both are directly regulated by the RegA and Ler regulatory proteins [83]. This then leads to greater induction of the *grlA* gene relative to *grlR* as its expression is being driven from both the *grlR* promoter and its own [83]. Given the proposal of TreR possessing activator activity (discussed below), this co-transcription may then amplify *treA* expression levels, similar to the positive autoregulatory effect of DdlR which activate the *ddl* and *ddlR* genes involved in peptidoglycan biosynthesis [84].

Canonically, TreR negatively regulates the expression of trehalose metabolism genes [32, 85], therefore we sought to understand its regulatory action in *C. difficile* using a mechanistic approach. In particular, the TreR protein within RT027 strains was thought to enhance sensitivity towards trehalose and increase *treA* expression due to a L172I amino acid substitution [18]. Firstly, we identified putative TreR binding sites within the promoter regions of both the *treR* and *treA* genes which were consistent with sequences of other known HutC subfamily regulators, such as the *C. difficile* CelR protein that regulates cellobiose metabolism [17]. Next, we showed the R20291 TreR regulatory protein can bind to both the *treR* and *treA* gene promoter regions *in vitro* (Fig. 5a, b). Interestingly, TreR may regulate its own expression due to the presence of a putative TreR binding site in its promoter region, and similar observations have been found for the *C. difficile celR* gene which possesses a CelR binding site in its promoter region which also exhibits autoregulatory activity [17]. Although not mechanistically investigated in this work as we used a recombinant R20291 TreR protein, the M120 *ptsT* and *treX* genes possessed putative TreR binding sites in their upstream regions, indicating these genes are likely targets for TreR regulation. Finally, trehalose-6-phosphate and not trehalose was identified as the effector of TreR, presumably due to the PTS-mediated transport of trehalose into the cell, as shown for the *B. subtilis* TreR protein [26, 32]. As the presence of trehalose-6-phosphate was observed to increase the amount of shifted DNA at lower TreR concentrations, this provides evidence that an activator function may be present. Similar observations of increased DNA binding ability have been observed for another GntR regulator, CitO, in *Enterococcus faecalis* which activates citrate fermentation genes [86, 87]. Additionally, TreR has been shown to activate the expression of trehalose metabolism genes within *Streptococcus mutans *, whereby the deletion of the *treR* gene prevents expression of *treA* and *treB* and causes an *in vitro* growth defect [34]. Although we described the interaction of TreR, its target genes and effector, this provides only mechanistic information and not the exact mechanism of signal transduction to regulate gene expression. Further work involving mutation of the *treR* gene would prove useful in determining its true regulatory function as a repressor or activator, which was unattainable in this current work. This would then provide the precise regulatory actions involved in the regulation of *C. difficile* trehalose metabolism.

Finally, we determined if trehalose modulated the gene transcription of processes involved in colonisation and/or pathogenesis given its implication in virulence [18]. RNA-seq of *C. difficile* R20291 cultured in trehalose revealed that the most highly expressed genes, *CDR20291_2971*, *pgmB* and *CDR20291_2973*, may constitute a previously unidentified trehalose metabolism pathway based on similarities to this genetic locus in *L. lactis* [56]. Interestingly, *CDR20291_2971*, *pgmB* and *CDR20291_2973* cannot be found in clade three and five strains, probably due to the presence of the SGC. As no annotated trehalose-specific PTS gene exists within strains belonging to clades one, two and four, the *CDR20291_2971* gene represents a candidate to allow trehalose transport. Another gene which could be a candidate for this process is *CDR20291_2928* (2.24 log_2_ fold change), a putative PTS system IIABC component gene which is immediately upstream of the *treR* gene. To support this, a 630 isolate which possesses an E258D amino acid substitution within the equivalent *CD630_30890* gene has been shown to facilitate increased growth in 10 mM trehalose, implicating this gene in trehalose transport [62]. However, deletion of *treA* in the R20291 strain has been shown to abolish growth in 10 mM trehalose, therefore these three genes may be insufficient to allow increased growth [18]. Instead, they may constitute cryptic genes, only showing a growth phenotype following mutation of *cis* regulatory elements and/or *trans*-acting factors [88, 89]. The function of these genes will require scrutinising to determine if there are any involvement, if at all, in mediating the transport and metabolism of trehalose in *C. difficile *.

Our work here demonstrates the genetic variability of trehalose metabolism genes that exist in *C. difficile* and how these differences influence growth across the representative strains used. These findings expand our understanding of the molecular basis of trehalose metabolism in *C. difficile *, however mutant studies would bolster this knowledge further to elucidate the exact mechanisms to allow this process. These include the regulatory activity of TreR and the determination of the trehalose-specific PTS which permits the intake of trehalose in those strains belonging to clade one, two and four, such as the *CDR20291_2971* and *CDR20291_2928* genes which were identified by RNA-seq. We show that toxin and sporulation gene expression are lowered in the hypervirulent R20291 and M120 strains when cultured in trehalose, however how these *in vitro* findings translate to an *in vivo* context requires further study. These data provide further information into understanding the role of individual metabolites which *C. difficile* utilise for growth, showcasing that trehalose metabolism is variably metabolised and further research is required to understand this further.

## Supplementary Data

Supplementary material 1
