# Supplementary material for: One size does not fit all – Trehalose metabolism by Clostridioides difficile is variable across the five phylogenetic lineages
Source: Microb Genom. 2023 Sep 28;9(9):001110. doi: 10.1099/mgen.0.001110 (PMC10569727; doi:10.1099/mgen.0.001110)
Supplement: Supplementary material 1 [file mgen-9-1110-s001.pdf]

Supplemental material

One size does not fit all – Trehalose metabolism by *Clostridioides difficile* is variable across the five phylogenetic lineages

Andrew Marshall, John W. McGrath, Molly Mitchell, Séamus Fanning, Geoff McMullan

Fig. S1. The *treR*-*treA* and *ptsT*-*treA2* genes are co-transcribed as an operon.

Fig. S2. Putative TreR bindings sites can be located within the promoter regions of the *treR* and *treA* genes.

Table S1: Oligonucleotide sequences used in this study.

Table S2. List of differentially expressed genes with a fold change greater than 2 or less than -2 in the *C. difficile* R20291 strain during growth in 10 mM trehalose relative to growth in 20 mM glucose.

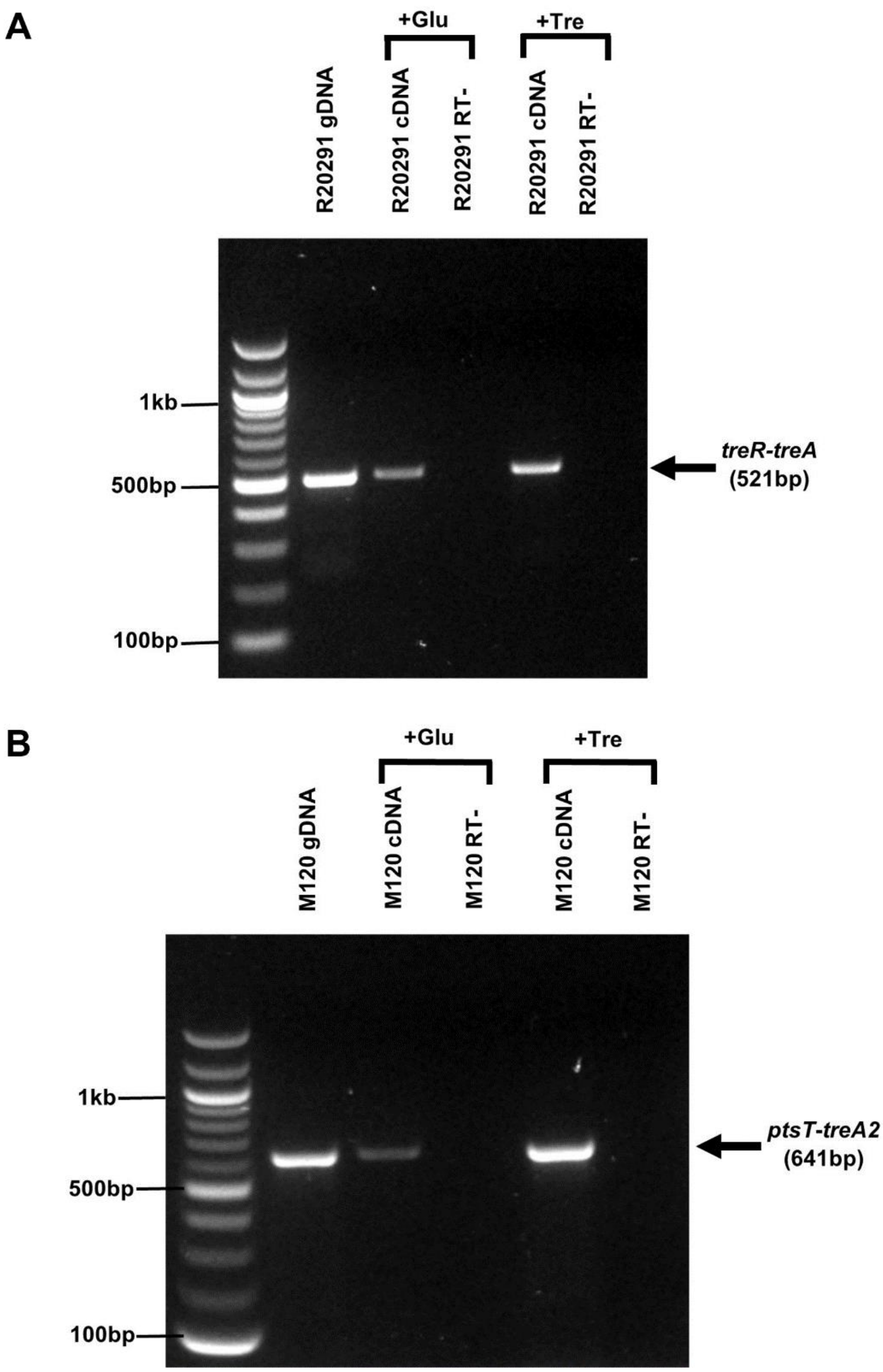

Fig. S1. The *treR-treA* and *ptsT-treA2* genes are co-transcribed as an operon. Cultures of *C. difficile* R20291 and M120 were grown in the presence of 20 mM glucose or 10 mM trehalose, harvested for RNA extraction and processed for cDNA generation as described in Materials and Methods. PCR was performed using genomic DNA (gDNA, positive control), cDNA or the no reverse transcriptase control (RT-, negative control). Products were generated using primers that amplified overlapping regions of the (A) 3’ end of the *treR* gene and 5’ end of the *treA* gene and (B) 3’ end of the *ptsT* gene and 5’ end of the *treA2* gene. 30 cycles were carried out for each and products resolved on a 2% agarose gel, arrows indicate expected product size.

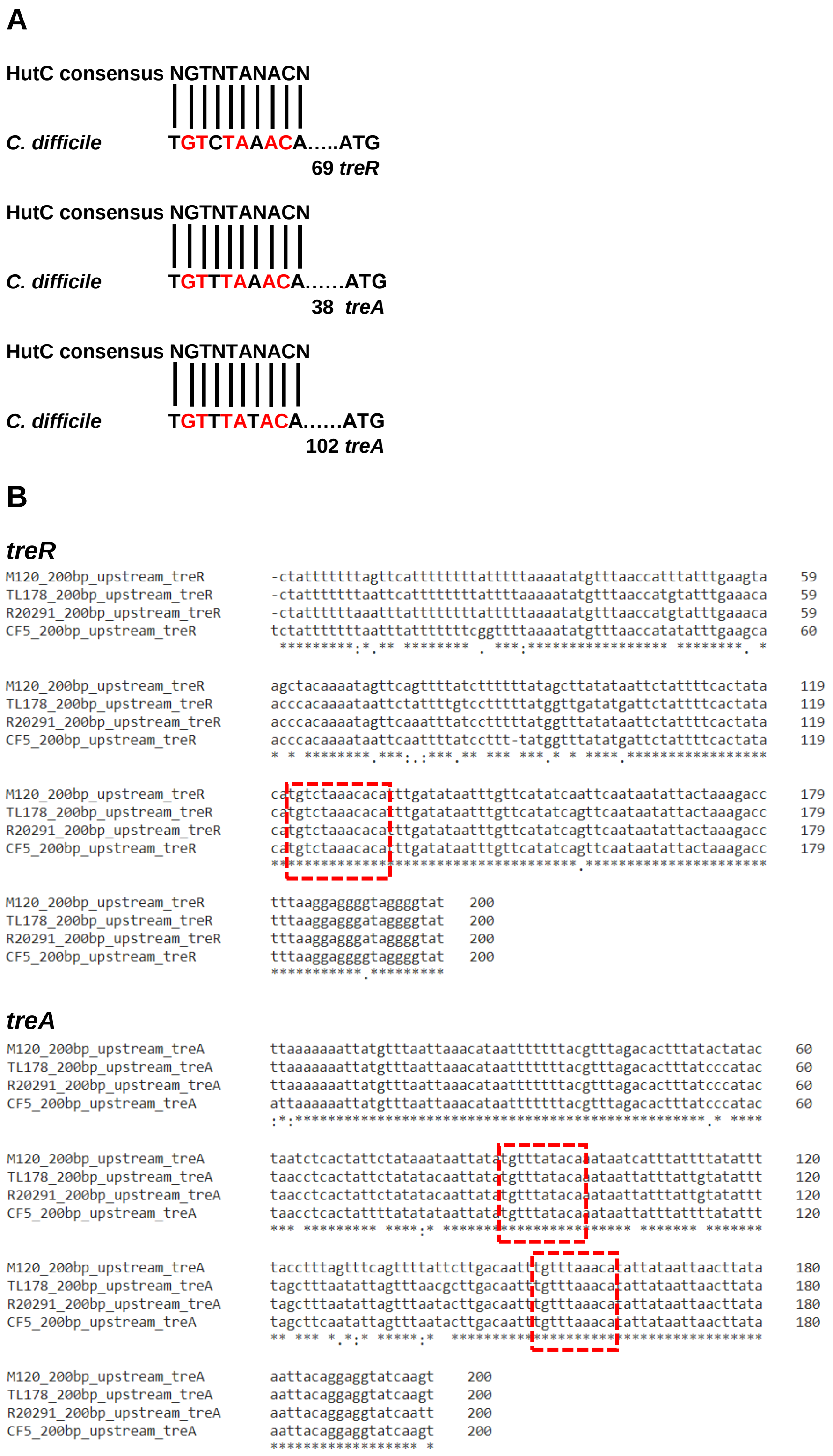

Fig. S2. Putative TreR bindings sites can be located within the promoter regions of the *treR* and *treA* genes. (A) Alignments of the putative TreR binding sites in the promoter region of the *C. difficile treR* and *treA* genes as compared to the HutC subfamily regulator consensus sequence. (B) Clustal Omega alignments of the 200 bp upstream promoter region of the M120, TL178, R20291 and CF5 *treR* and *treA* genes. Red dashed boxes indicate putative TreR binding sites. Asterisks (*) denote identical nucleotides, colons (:) indicate similar residues and dots (.) indicate low similarity.

Table S1: Oligonucleotide sequences used in this study. 'F' and 'R' denotes forward and reverse primer respectively.

Gene	Description	Sequence (5'-3')	Product Size (bp)	Annealing Temperature (^oC)	Reference
**Reverse transcription-PCR**					
treR-treA	3' end of *treR* gene to 5' end of *treA* gene	F-AGAGCTTGAGTTAAAAACGCTACC<br>R-AGGAGAAACGTACATAGGAGTCA	521	60	This study
ptsT-treA2	3' end of *ptsT* gene to 5' end of *treA2* gene	F-AGCTACAGCTGTTGGGGTTG<br>R-TCCCAATTTGTTGGAGCACT	641	56.7	This study
**Quantitative PCR**					
treA	Phosphotrehalase	F-TACGCTGATGGTCCTCGTAT<br>R-CGCCTCCTTTATAATCTGTTTTC	173	60	[1]
treR	Trehalose repressor protein	F-GCAAGGGTGTTGTAGTGTTAGA<br>R-AGTGGAGAACACTAGTAGCAGTA	126	60	This study
ptsT	Phosphotransferase system EIIBC trehalose component	F-TCAGCATGGCTAGGAATAACAG<br>R-TCCTATTGCACTTGCTACCATAA	96	60	This study
treA2	Putative phosphotrehalase orthologue	F-ACTTAATGAGTGGCAAATAGGAATG<br>R-CGTGGTTGGTCATGGTTACA	78	60	This study
treX	Putative trehalase enzyme	F-ATGGAGTTGGGATCACTGTTT<br>R-TGACCATATTCATCTTGGTAGTCG	108	60	This study
treR2	Putative trehalose repressor protein orthologue (*C. difficile* M120)	F-GAGCTATACAGGAATTAGCAAACC<br>R-AAACTCTGATTGCTTGTTAGCC	95	60	This study
treR2	Putative trehalose repressor protein orthologue (*C. difficile* CD305)	F-CAGGAATTAGCAAACCAAGGATATG<br>R-AACTCTGATTGTGTATTAGCCCTAT	86	60	This study
tcdA	Toxin A	F-GTCGGATTGCAAGTAATTGACAATA<br>R-TAACAGTCTGCCAACCTTTTGAGA	140	60	[2]
tcdB	Toxin B (*C. difficile* R20291 RT027)	F-CACTTCTTTTCAGCACCATCA<br>R-CTGGTGTCCATCCTGTTTCC	160	60	[2]
tcdB	Toxin B (*C. difficile* M120 RT078)	F-TGGCTGAAGCTAATGCAGATAA<br>R-CATCTGGGAAGAAACCAACTCT	78	60	This study
sigE	Early sporulation sigma factor E	F-GATTTGTTGGTTATGGCACTTGA<br>R-GTAGGACTGTGATATTCCAAGCA	138	60	This study
rpoA	RNA polymerase subunit alpha	F-TTTGACCAACTCTTGTGTTTTCC<br>R-TAAAGGTAGAGGTTATGTTTCTGCT	133	60	[2]
tpi	Triose phosphate isomerase	F-GCAGGAAACTGGAAAATGCATAA<br>R-CAGATTGGCTCATATGCAACAAC	488	50	[3]

**Electrophoretic mobility shift assay**					
treA Promoter	Phosphotrehalase promoter region Biotinylated	F-GCTTGAGTTAAAAACGCTACCT<br>R-GGAGAAACGTACATAGGAGTCA	517	63	This study
treR Promoter	Trehalose repressor protein promoter region Biotinylated	F-TGGCACCAGCATAGTCTTTCC<br>R-CCCTATCCCTCCTTAAAGGTCT	431	63.6	This study

Table S2. List of differentially expressed genes with a fold change greater than 2 or less than -2 in the *C. difficile* R20291 strain during growth in 10 mM trehalose relative to growth in 20 mM glucose.

Locus name	Gene name	Gene description	Fold change (log_2) Trehalose vs glucose
Upregulated genes			
CDR20291_2972	*pgmB*	beta-phosphoglucomutase	5.82
CDR20291_2974	*acsB3*	putative 6-phospho-beta-glucosidase	5.78
CDR20291_2930	*treA*	trehalose-6-phosphate hydrolase	5.58
CDR20291_2973	*CDR20291_2973*	putative glycosyl hydrolase	5.48
CDR20291_2971	*CDR20291_2971*	PTS system, IIabc component	5.07
CDR20291_0728	*CDR20291_0728*	Putative hydroxymethylglutaryl-CoA lyase	4.61
CDR20291_2241	*grdE*	glycine reductase complex component B alpha and beta subunits	4.22
CDR20291_2239	*grdB*	glycine reductase complex component B gamma subunit	4.03
CDR20291_2240	*grdA*	glycine/sarcosine/betaine reductase complex component A	4.02
CDR20291_2238	*grdC*	glycine/sarcosine/betaine reductase complex component C beta subunit	3.74
CDR20291_2242	*trxA2*	Thioredoxin	3.39
CDR20291_2237	*grdD*	glycine/sarcosine/betaine reductase complex component C alpha subunit	3.39
CDR20291_2243	*trxB3*	thioredoxin reductase	3.36
CDR20291_2292	*CDR20291_2292*	putative cell wall hydrolase	3.28
CDR20291_2244	*grdX*	putative glycine reductase complex component	3.19
CDR20291_0729	*CDR20291_0729*	putative membrane protein	3.09
CDR20291_1521	*CDR20291_1521*	putative nitric oxide reductase flavoprotein	2.94
CDR20291_2788	*ntpB*	V-type sodium ATP synthase subunit B	2.73
CDR20291_2373	*CDR20291_2373*	putative exported protein	2.69
CDR20291_2787	*ntpD*	V-type ATP synthase subunit D	2.66
CDR20291_2791	*ntpC*	V-type ATP synthase subunit C	2.62
CDR20291_2569	*CDR20291_2569*	putative calcium-chelating exported protein	2.59
CDR20291_2790	*ntpG*	V-type sodium ATP synthase subunit G	2.59
CDR20291_2792	*ntpE*	V-type sodium ATP synthase subunit E	2.46
CDR20291_2687	*CDR20291_2687*	cell surface protein	2.46
CDR20291_2789	*ntpA*	V-type ATP synthase subunit A	2.43
CDR20291_0507	*gapN*	NADP-dependent glyceraldehyde-3-phosphate dehydrogenase	2.42
CDR20291_0730	*act*	putative beta-alanine CoA-transferase	2.40
CDR20291_2793	*ntpK*	V-type ATP synthase subunit K	2.38
CDR20291_2927	*CDR20291_2927*	putative cellobiose-phosphate degrading protein	2.34
CDR20291_3203	*CDR20291_3203*	ABC transporter, ATP-binding protein	2.26
CDR20291_2928	*CDR20291_2928*	PTS system, IIabc component	2.24
CDR20291_2672	*CDR20291_2672*	cell surface protein	2.19
CDR20291_1492	*cysM*	putative O-acetylserine sulfhydrylase	2.13
CDR20291_2026	*thrB*	homoserine kinase	2.11
CDR20291_0210	*CDR20291_0210*	putative sugar-phosphate kinase	2.11

CDR20291_1493	*cysA*	serine acetyltransferase	2.09
CDR20291_0758	*CDR20291_0758*	putative oxidative stress protein	2.06
CDR20291_3449	*CDR20291_3449*	putative glyoxalase	2.04
CDR20291_0206	*CDR20291_0206*	putative transcription antiterminator	2.02
Downregulated genes			
CDR20291_1239	*CDR20291_1239*	Putative membrane protein	-2.00
CDR20291_2552	*murE*	putative UDP-N-acetylmuramoylalanyl-D-glutamate--2,6-diaminopimelate ligase	-2.01
CDR20291_2557	*CDR20291_2557*	putative Na(+)/H(+) antiporter	-2.03
CDR20291_0865	*CDR20291_0865*	GntR-family transcriptional regulator	-2.04
CDR20291_2435	*CDR20291_2435*	putative ABC sugar transporter, permease protein	-2.05
CDR20291_3478	*CDR20291_3478*	transposase-like protein b	-2.05
CDR20291_2315	*CDR20291_2315*	putative aminotransferase	-2.06
CDR20291_0859	*CDR20291_0859*	Conserved hypothetical protein	-2.07
CDR20291_2436	*CDR20291_2436*	putative ABC sugar transporter, permease protein	-2.09
CDR20291_0746	*CDR20291_0746*	probable transporter (MFS)	-2.11
CDR20291_0858	*CDR20291_0858*	NAD-dependent malic enzyme	-2.12
CDR20291_0363	*CDR20291_0363*	Radical SAM-superfamily protein	-2.13
CDR20291_0287	*CDR20291_0287*	PTS system, IIa component	-2.14
CDR20291_2556	*licT*	putative transcription antiterminator	-2.15
CDR20291_2437	*CDR20291_2437*	putative sugar transporter, substrate-binding lipoprotein	-2.18
CDR20291_2559	*appD*	oligopeptide transporter, ATP-binding protein	-2.18
CDR20291_1876	*CDR20291_1876*	conserved hypothetical protein	-2.19
CDR20291_2482	*pyrR*	bifunctional protein PyrR	-2.20
CDR20291_2773	*fhuB*	putative ferrichrome ABC transporter, permease protein	-2.23
CDR20291_1487	*CDR20291_1487*	putative ribose ABC transporter, substrate-binding lipoprotein	-2.25
CDR20291_0288	*CDR20291_0288*	PTS system, IIb component	-2.27
CDR20291_1546	*CDR20291_1546*	putative iron compound ABC transporter, permease protein	-2.27
CDR20291_2434	*CDR20291_2434*	conserved hypothetical protein	-2.29
CDR20291_2108	*CDR20291_2108*	lactose permease	-2.29
CDR20291_1545	*CDR20291_1545*	putative iron compound ABC transporter, permease protein	-2.31
CDR20291_2871	*CDR20291_2871*	proton-dependent oligopeptide transporter	-2.34
CDR20291_0516	*CDR20291_0516*	putative cation transporting ATPase	-2.35
CDR20291_0856	*fumA*	fumarate hydratase, subunit A	-2.38
CDR20291_1328	*feoB1*	Ferrous iron transport protein B	-2.38
CDR20291_1329	*CDR20291_1329*	putative exported protein	-2.39
CDR20291_2213	*gatD*	putative galactitol-1-phosphate 5-dehydrogenase	-2.41
CDR20291_2350	*CDR20291_2350*	conserved hypothetical protein	-2.42
CDR20291_0364	*CDR20291_0364*	putative membrane protein	-2.43
CDR20291_2014	*CDR20291_2014*	putative xanthine/uracil permease	-2.44
CDR20291_0806	*CDR20291_0806*	ABC transporter, ATP-binding protein	-2.44
CDR20291_1560	*guaD*	guanine deaminase	-2.45
CDR20291_0803	*CDR20291_0803*	ABC transporter, ATP-binding protein	-2.45
CDR20291_2774	*fhuD*	putative ferrichrome ABC transporter,substrate-binding protein	-2.52
CDR20291_0807	*CDR20291_0807*	abc transporter, permease protein	-2.52

CDR20291_0289	*CDR20291_0289*	putative PTS system, IIa component	-2.53
CDR20291_1639	*CDR20291_1639*	putative ferrous iron transport protein A	-2.53
CDR20291_0666	*CDR20291_0666*	putative exported protein	-2.54
CDR20291_0802	*CDR20291_0802*	ABC transporter, substrate-binding lipoprotein	-2.60
CDR20291_0804	*CDR20291_0804*	ABC transporter, permease protein	-2.62
CDR20291_1326	*CDR20291_1326*	putative ferrous iron transport protein	-2.62
CDR20291_2057	*CDR20291_2057*	putative membrane protein	-2.72
CDR20291_0134	*CDR20291_0134*	PTS system, IIa component	-2.75
CDR20291_0136	*CDR20291_0136*	PTS system, IIc component	-2.80
CDR20291_2055	*CDR20291_2055*	putative exported protein	-2.82
CDR20291_2416	*CDR20291_2416*	conserved hypothetical protein	-2.85
CDR20291_2349	*CDR20291_2349*	ABC transporter, ATP-binding protein	-2.93
CDR20291_1327	*feoA1*	putative ferrous iron transport protein A	-3.05
CDR20291_1275	*CDR20291_1275*	hypothetical protein	-3.07
CDR20291_2554	*crr*	PTS system, glucose-specific IIa component	-3.08
CDR20291_0137	*CDR20291_0137*	conserved hypothetical protein	-3.10
CDR20291_2555	*ptsG*	PTS system, glucose-specific IIbc component	-3.13
CDR20291_1925	*fldX*	Flavodoxin	-3.15
CDR20291_0146	*CDR20291_0146*	putative riboflavin transporter	-3.17
CDR20291_0215	*purE*	phosphoribosylaminoimidazole carboxylase catalytic subunit	-3.31
CDR20291_2487	*CDR20291_2487*	putative carbon starvation	-3.37
CDR20291_2417	*CDR20291_2417*	conserved hypothetical protein	-3.43
CDR20291_0432	*CDR20291_0432*	putative phosphoribosylaminoimidazole-succinocarboxamide synthase	-3.47
CDR20291_0216	*purC*	phosphoribosylaminoimidazole-succinocarboxamide synthase	-3.48
CDR20291_2418	*CDR20291_2418*	putative membrane protein	-3.66
CDR20291_0517	*CDR20291_0517*	putative membrane protein	-3.67
CDR20291_0217	*purF*	amidophosphoribosyltransferase	-3.69
CDR20291_0222	*purL*	formylglycinamidine ribonucleotide synthetase	-3.72
CDR20291_1255	*ddl*	D-alanine--D-alanine ligase B	-3.74
CDR20291_2391	*CDR20291_2391*	hypothetical protein	-3.84
CDR20291_2419	*CDR20291_2419*	putative aminotransferase	-3.84
CDR20291_0135	*CDR20291_0135*	PTS system, IIb component	-3.86
CDR20291_0218	*purG*	phosphoribosylformylglycinamidine cyclo-ligase	-3.87
CDR20291_1334	*CDR20291_1334*	conserved hypothetical protein	-4.03
CDR20291_2937	*CDR20291_2937*	putative amidohydrolase	-4.07
CDR20291_2938	*CDR20291_2938*	putative C4-dicarboxylate anaerobic carrier	-4.30
CDR20291_0219	*purN*	phosphoribosylglycinamide formyltransferase	-4.34
CDR20291_0221	*purD*	phosphoribosylamine--glycine ligase	-4.48
CDR20291_0220	*purH*	bifunctional purine biosynthesis protein	-4.52
CDR20291_1598	*ribD*	riboflavin biosynthesis protein	-6.74
CDR20291_1597	*ribB*	riboflavin synthase alpha chain	-8.21
CDR20291_1596	*ribA*	riboflavin biosynthesis protein	-9.84
CDR20291_1595	*ribH*	riboflavin synthase beta chain	-10.09

References:

1. Collins J, Robinson C, Danhof H, Knetsch CW, van Leeuwen HC, *et al.* Dietary trehalose enhances virulence of epidemic Clostridium difficile. *Nature* 2018;553:291–294.

2. Barketi-Klai A, Monot M, Hoys S, Lambert-Bordes S, Kuehne SA, *et al*. The flagellin FliC of Clostridium difficile is responsible for pleiotropic gene regulation during in vivo infection. *PLoS One* 2014; 19;9(5):e96876.

3. Lemee L, Dhalluin A, Testelin S, Mattrat MA, Maillard K, *et al*. Multiplex PCR targeting tpi (triose phosphate isomerase), tcdA (Toxin A), and tcdB (Toxin B) genes for toxigenic culture of Clostridium difficile. *J Clin Microbiol*. 2004 Dec;42(12):5710-4.
